# Isolation and Characterisation of Bacteria From an Extremely High Boron and Salinity‐Tolerant *Puccinellia distans* (Jacq.) Parl. Rhizosphere and Their Potential Impact on the Growth of Bread Wheat ( *Triticum aestivum* L.)

**DOI:** 10.1111/1758-2229.70105

**Published:** 2025-09-10

**Authors:** Noyan Eken, Ozgur Ates, Ramazan Cakmakci, Ummahan Cetin Karaca, Sait Gezgin, Erdogan Esref Hakki

**Affiliations:** ^1^ Department of Organic Agriculture Program, Odemis Vocational School Ege University Izmir Türkiye; ^2^ Department of Soil Science and Plant Nutrition, Faculty of Agriculture Selcuk University Konya Türkiye; ^3^ Transitional Zone Agricultural Research Institute Eskisehir Türkiye; ^4^ Department of Field Crops, Faculty of Agriculture Canakkale Onsekiz Mart University Canakkale Türkiye

**Keywords:** boron toxicity, bread wheat, extremophilic bacteria, molecular characaterization, plant growth‐promoting rhizobacteria (PGPR)

## Abstract

Boron toxicity and salinity are major abiotic stress factors that cause significant yield losses, particularly in arid and semi‐arid regions. Hyperaccumulator plants, such as *Puccinella distans* (Jacq.) Parl. from Kirka, exhibit remarkable tolerance to these stresses. This study isolated nine boron‐tolerant and six salinity‐tolerant bacterial strains from the rhizosphere of *P. distans* . Using BLAST analysis of 16S rRNA sequences, the boron‐tolerant bacteria were identified as *Sphingobacterium detergens* (three strains), *Achromobacter spanius* , *Pseudomonas extremorientalis* , *Olivibacter soli* , *Puccinella poae* , *Zhihengliuella halotolerans* and *Chryseobacterium* sp., while the salinity‐tolerant strains included *Bacillus toyonensis*, *B. muralis* , *Staphylococcus warneri* , *Staphylococcus aureus* (two strains) and *Arthrobacter aurescens* . Among these, *P. poae* stood out as a promising plant growth‐promoting rhizobacteria (PGPR) candidate. A greenhouse pot experiment demonstrated that inoculating *Triticum aestivum* L. with *P. poae* under varying boron doses significantly enhanced plant growth. Notable increases were observed in plant height, root length, stem fresh weight and emergence ratio. These findings highlight the potential of *P. poae* as an eco‐friendly microbial fertiliser to enhance crop resilience in boron‐affected areas and offer a sustainable alternative to chemical fertilisers, thus improving agricultural productivity under abiotic stress conditions.

## Graphical abstract

Under greenhouse conditions, the boron‐tolerant bacterium *Pseudomonas poae* demonstrated a significant enhancement in wheat growth under boron‐induced stress. These findings highlight its promising role as a sustainable biofertiliser to bolster crop productivity in boron‐affected agroecosystems.

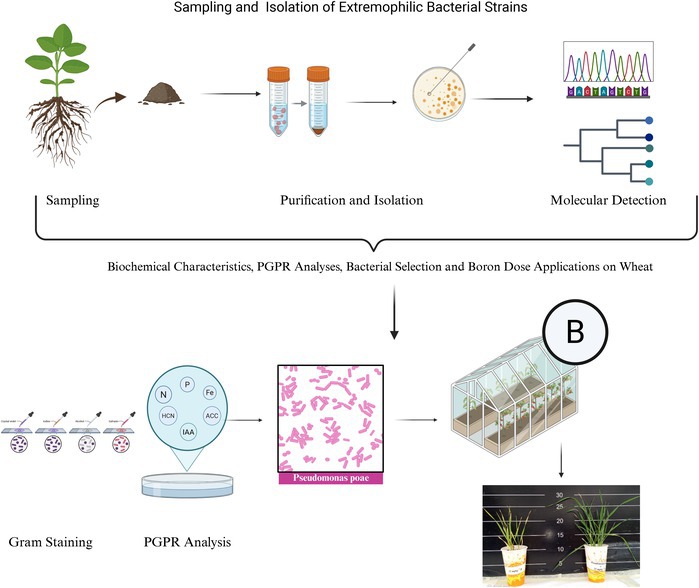

## 1 Introduction

Boron toxicity and soil salinisation are among the most critical abiotic stressors impacting global agriculture, particularly, in arid and semi‐arid regions. Symptoms of boron toxicity vary between species with limited and significant phloem mobility. In phloem‐motile species, boron moves through the xylem and accumulates at the end of the transpiration stream. Accordingly, leaf symptoms include chlorosis and necrosis spreading from the leaf tips, with brown lesions first forming on the margins and later covering most of the leaf surface. The oldest leaves are the first to be affected by the disorder, which then spreads to the top of the plant (Torun et al. 2006; Brdar‐Jokanović et al. 2013). These conditions not only reduce crop productivity but also contribute to long‐term environmental degradation, threatening food security and sustainable land management. With the exacerbating effects of global climate change, addressing soil salinization has become a priority for researchers and policymakers worldwide (Kopittke et al. 2019; Malhi et al. 2020). In this context, cultivating salt‐ and boron‐tolerant plant species has emerged as a promising strategy to restore degraded lands and improve soil health. The bacterial community was more sensitive to boron, and high boron content caused toxicity in both the microbial community and plants (Vera et al. 2021). Halophytes such as *Tripolium pannonicum, Suaeda glauca, Iris wilsonii, Puccinella tenuiflora* and *Echinochloa frumentacea* have demonstrated remarkable resilience to extreme boron toxicity and salinity, making them valuable for ecological restoration efforts. These species not only thrive under challenging conditions but also ameliorate soil properties by reducing pH and total salt content while increasing organic matter (Zhao et al. 2019; Cheng et al. 2023).

In addition to boron toxicity, alkalinity and salinity‐tolerant plants, arbuscular mycorrhizal fungi (AMF) provide a sustainable solution to these stresses (Valsalakumar et al. 2007; Yang et al. 2020). In parallel, plant growth‐promoting rhizobacteria (PGPR) have attracted considerable attention for their ability to enhance plant growth and improve stress tolerance. PGPR contribute to plant health through diverse mechanisms, including nitrogen fixation, phosphate solubilisation and the production of phytohormones such as indole‐3‐acetic acid (IAA) and cytokinins (Jha and Saraf 2015; Agami et al. 2017). For instance, *Bacillus* and *Paenibacillus* species have been shown to improve nutrient availability, stimulate root and shoot development and mitigate oxidative stress in plants exposed to salinity and alkalinity (de Freitas et al. 1997; Zhang et al. 2020). Recent research highlights the synergistic effects of combining AMF and PGPR, amplifying their benefits for plant growth and soil remediation (Bishnoi 2015; Khan et al. 2016). Among halophytes, *Puccinella distans* (barren grass) stands out due to its exceptional tolerance to saline and alkaline soils, as well as its capacity to thrive in boron‐rich environments (Hamurcu et al. 2016; Hakki et al. 2019). This species is naturally found in the saline and boron‐rich soils of Türkiye, particularly, around salt lakes and boron mines (Hakki et al. 2019). *P. distans* has been shown to endure high levels of boron stress, employing antioxidant defence mechanisms to mitigate its toxic effects (Hamurcu et al. 2016). However, while studies have extensively documented its physiological responses to abiotic stress, less is known about the microbial communities associated with its rhizosphere and their potential roles in enhancing plant tolerance.

Bacteria capable of thriving under extreme conditions, termed extremophiles, are of particular interest in this context. Extremophilic bacteria, such as those isolated from boron‐ and salt‐rich soils, exhibit unique adaptations that enable them to survive in harsh environments. For instance, bacterial genera such as *Arthrobacter*, *Rhodococcus* and *Bacillus* have been identified in boron‐rich soils of Türkiye (Ahmed and Fujiwara 2010), while halotolerant species like *Gracilibacillus* and *Halobacterium* have been isolated from saline environments (Sorokin et al. 2006; Baati et al. 2010). These bacteria often possess plant growth‐promoting traits, such as nitrogen fixation and phosphate solubilisation, making them valuable for sustainable agriculture in degraded soils (Timmusk et al. 1999; Jha et al. 2015). This study aims to explore the plant growth‐promoting potential of bacteria isolated from the rhizosphere of *P. distans* , a halophyte native to boron‐and salt‐rich habitats. By characterising these bacteria's functional traits and phylogenetic relationships, this research seeks to advance our understanding of plant‐microbe interactions in extreme environments. The findings will contribute to the development of microbial‐based solutions for enhancing plant resilience and soil health in boron‐saline ecosystems, addressing the dual challenges of environmental restoration and agricultural sustainability (Chebotar et al. 2023; Eken and Hakki 2024). There is a very narrow range between both boron deficiency and toxicity that reduces crop yield and quality worldwide and deficiency problems can be solved by fertilisation, while soil boron toxicity can be ameliorated using various procedures; however, these approaches are costly, difficult, time‐consuming and often show temporary effects (Princi et al. 2015; Brdar‐Jokanović 2020). Therefore, it is important to investigate the possible relationship and connection between the differences in reaction and tolerance developed by species against the environments in which they grow, agronomic management, plant and rhizospheric bacteria and boron tolerance.

## 2 Materials and Methods

### 2.1 Sample Collection and Isolation of Bacteria

Soil samples were collected from the rhizosphere of *P. distans* (Jacq.) Parl., a plant highly tolerant to salinity and boron toxicity, at two distinct locations in Türkiye: Eskişehir Kırka Boron Mine (39°17′38.6″ N, 30°29′28.9″ E) and Central Anatolian Salt Lake (38°24′41″ N, 33°32′14″ E). Samples were transported to the laboratory at 4°C for further analysis. Rhizospheric bacteria were isolated using serial dilutions (10^−4^–10^−8^) prepared in sterile saline, followed by plating on Nutrient Agar (NA). Morphologically distinct colonies were purified using the streak plate method, resulting in 15 bacterial isolates, which were preserved at −80°C in Nutrient Broth (NB) supplemented with 20% glycerol. Additionally, *Escherichia coli* (ATCC 25922) and *Pantoea agglomerans* strains were included for comparative analysis (Gregory 2006). To isolate and obtain a pure single species/genus culture, we employed the streaking method, reducing the colonies to a single one. Fifteen colonies, identified as pure, were sent for molecular characterisation. Using this classical streaking technique, the 15 pure bacteria were confirmed by comparing the electropherograms from sequencing results verifying their purity.

### 2.2 Determination of Biochemical and PGPR Properties of Extremophilic Bacteria in the Presence of Reference Bacteria

#### 2.2.1 Biochemical Properties

Gram staining was performed on 18–24 h old bacterial cultures grown in NB to differentiate Gram‐positive and Gram‐negative bacteria based on cell wall properties. Gram‐positive bacteria retained the violet stain, while Gram‐negative bacteria appeared pink. Colony morphologies were subsequently analysed under a light microscope at ×100 magnification (Collee et al. 1996; Harley and Prescott 2002). The oxidase test was conducted using the oxidase test strips manufactured by Biotest Pharmaceutica in Dreieich, Germany. A sterile inoculation loop was used to transfer organisms from an 18 to 24 h old NA colony onto a microscope slide. A drop of 3% hydrogen peroxide (H_2_O_2_) was then added using a Pasteur pipette. Bubble formation indicated a positive oxidase reaction, whereas the absence of bubbles denoted a negative result (Chester 1979).

The urease test was performed using 18–24 h old bacterial cultures grown in NB. A loopful of inoculum was streaked onto sterilised urea agar medium and incubated at 37°C for 24–48 h. A positive result, indicating urea degradation into ammonia and carbon dioxide, was marked by the medium turning pink (Collee et al. 1996).

The citrate utilisation test was conducted using 18–24 h old bacterial cultures grown in NB. Bacterial colonies were inoculated onto Simmons citrate agar slants by stabbing the base and streaking the surface. After incubation at 37°C for 24–48 h, a positive result was indicated by an alkaline shift in the medium, demonstrating citrate utilisation and the production of alkaline by‐products during metabolism (Harley and Prescott 2002).

Fresh 18–24 h bacterial cultures grown in NB were inoculated into Triple Sugar Iron Broth (TSIB), which contains lactose, sucrose and glucose. The medium, initially red, turns yellow to indicate an acidic reaction from sugar fermentation, while an unchanged colour signifies an alkaline reaction, indicating no sugar fermentation. Black precipitate at the tube's base signifies hydrogen sulphide (H_2_S) production Sneath et al. (1951) and bubble formation indicates gas production (Collins et al. 2004). TSIB was prepared in 10 mL glass test tubes, incubated at 30°C for 5 days and evaluated for colour and gas changes at the end of the incubation period.

#### 2.2.2 PGPR Properties

In this study, the plant growth promoting properties of the isolated bacteria were determined by their nitrogen fixation, phosphate solubilisation, siderophore production, 1‐aminocyclopropane‐1‐carboxylate (ACC) deaminase activity, indole acetic acid production, HCN production and ammonia production properties.

Bacteria were inoculated onto NA using the line sowing technique from stock cultures. After 2–7 days of incubation, bacterial colonies were streaked onto nitrogen‐free Malate Sucrose Medium and incubated at 25°C–27°C for 7–10 days. Growth in the nitrogen‐free medium indicated nitrogen fixation, demonstrating the bacteria's ability to fix atmospheric nitrogen (Han et al. 2005).

Phosphate solubilisation by bacterial isolates was assessed following Mehta and Nautiyal (2001) methodology with modifications. Fresh 18–24 h bacterial cultures in NB were first grown in 5 mL of Luria‐Bertani (LB) medium for 24 h. Subsequently, 500 μL of the culture was inoculated into 50 mL of NBRIP‐BPB medium containing rock phosphate and tricalcium phosphate, with triplicates for each sample. Cultures were incubated at 28.5°C ± 1°C and agitated at 150 rpm for 5 days. After 120 h, supernatants were obtained via centrifugation at 15,000 rpm for 10 min and phosphorus content was analysed using ICP‐OES (Agilent 5110). Uninoculated NBRIP‐BPB medium served as a control to account for non‐bacterial phosphorus changes.

IAA production by bacterial isolates was assessed following the protocol of Bharucha et al. (2013). A 0.2 mL aliquot of a 24 h bacterial culture was inoculated into LB medium containing 0.2 mg mL^−1^ tryptophan and incubated at 28.5°C ± 1°C with 150 rpm agitation for 4 days. The experiment was performed in triplicate. After incubation, cultures were centrifuged at 15,000 rpm for 10 min at 4°C. IAA production was measured by mixing 1 mL of the supernatant with 4 mL of Salkowski reagent and allowing the mixture to develop a pink colour in the dark for 30 min. The colour intensity was measured at 535 nm using a UV/Visible spectrophotometer and IAA concentration was determined using a standard curve, expressed in μg mL^−1^ (Sarker and Al‐Rashid 2013).

The siderophore‐producing ability of bacterial strains was evaluated using the Chrome Azurol S (CAS) assay, following metodology Schwyn and Neilands' methodology (1987) with modifications. Glassware was pre‐washed with 3 mol L^−1^ hydrochloric acid (HCl) to remove potential iron residues, then rinsed with deionised water as described by (Cabaj and Kosakowska 2009). The CAS reagent was prepared by dissolving 121 mg of CAS in 100 mL distilled water, adding 20 mL of 1 mM ferric chloride solution and combining it with 20 mL of 10 mM HDTMA solution, as outlined by Schwyn and Neilands (1987). This mixture was sterilised before use. CAS agar plates were prepared by mixing 100 mL of CAS reagent with 900 mL LB agar medium. Bacterial strains were spot‐inoculated onto the plates, with an uninoculated control. After incubation at 28°C for 5–7 days, the presence of an orange zone around colonies indicated siderophore production (Louden et al. 2011).

The ACC deaminase activity of a selected bacterial isolate was evaluated using freshly grown bacterial cultures in 18–24 h of NB, based on the bacterium's ability to utilise ACC as a nitrogen source, following Penrose and Glick (2003). A 10 μL inoculum of a 24 h bacterial culture was streaked onto solid DF minimal medium containing 3.0 mM ACC Dworkin and Foster (1958). The plates were incubated at 28.5°C ± 1°C for 72 h. Positive ACC deaminase activity was indicated by bacterial growth on the ACC‐containing medium after the incubation period, demonstrating the bacterium's ability to utilise ACC as a nitrogen source.

Freshly grown bacterial cultures (18–24 h) in NB were used to assess NH_3_ (Ammonia) production, following the method described by Cappuccino and Sherman (1996). A 50 μL bacterial suspension was inoculated into 5 mL of peptone broth and incubated at 28°C for 48–72 h. After incubation, 250 μL of Nessler's reagent was added to each tube. The appearance of a brown–orange colour indicated ammonia production, confirming that the bacterial isolates produced ammonia as a metabolic byproduct during growth in the peptone broth.

Freshly grown bacterial cultures (18–24 h) in NB were used to evaluate HCN (hydrogen cyanide) production, following the protocol described by (Bakker and Schippers 1987). Bacterial isolates were streaked on King's B medium supplemented with 4.4 g L^−1^ glycine. A sterile filter paper, saturated with a picric acid solution (2.5 g of picric acid and 12.5 g of Na_2_CO_3_ in 1000 mL distilled water), was placed in the upper lid of the Petri plate. The plates were sealed with Parafilm to prevent gas leakage and incubated at 28°C for 48 h. During incubation, HCN produced by the bacteria diffused and interacted with the picric acid, causing a colour change. A yellow‐to‐light‐brown colour indicated a weak (+) reaction, brown indicated a moderate (++) reaction and reddish‐brown indicated a strong (+++) reaction.

### 2.3 Molecular Characterisation

#### 2.3.1 DNA Extraction and PCR Amplification

Genomic DNA was isolated using the EurX GeneMATRIX Bacterial & Yeast Genomic DNA Purification Kit (Poland). The DNA purity and quantity were assessed using the Thermo Scientific NanoDrop 2000 (USA) and the samples were visualised on a 1% agarose gel in 1X TBE buffer. PCR amplification of the 16S rRNA gene was performed using two universal primer pairs: 16S Forward (AGA GTT TGA TCC TGG CTC AG) and 16S Reverse (ACG GCT ACC TTG TTA CGA CTT) for the first pair and 27F (AGA GTT TGA TCM TGG CTC AG) and 1492R (TAC GGY TAC CTT GTT ACG ACT T) for the second. The final PCR reaction volumes were 20 μL for the first pair and 35.32 μL for the second. The PCR protocol for the first primer pair involved an initial denaturation at 95°C for 30 s, followed by 34 cycles of denaturation at 95°C for 1 min, annealing at 56°C for 30 s and elongation at 68°C for 90 s. For the second primer pair, the protocol included an initial denaturation at 95°C for 5 min, followed by 40 cycles of denaturation at 95°C for 45 s, annealing at 57°C for 45 s and elongation at 72°C for 5 min. PCR products were purified using the MAGBIO HighPrep PCR Clean‐up System (AC‐60005) and subsequently sequenced in both directions at BM Labosis (Ankara, Türkiye) using the ABI 3730XL Sanger sequencer (Applied Biosystems) and BigDye Terminator v3.1 Cycle Sequencing Kit (Applied Biosystems).

#### 2.3.2 Construction of Phylogenetic Trees

The phylogenetic tree representing the relationships among 15 isolated bacterial strains and 2 reference strains ( *E. coli* ATCC 25922 and *P.agglomerans* ) was constructed using the BLAST algorithm from the NCBI database. The genetic data for *E. coli* (ATCC 25922) were obtained from the genome available in the ATCC database, while the sequence data for *P. agglomerans* were derived from sequencing efforts. Genome sequences were aligned using the BioEdit bioinformatics programme, followed by Multiple Sequence Alignment (MSA) analysis. The MSA results were used to construct the phylogenetic tree using MAFFT 7 software with the neighbour‐joining method, 1000 bootstrap replicates and the Jukes–Cantor model. The phylogenetic tree revealed a 1% sequence divergence among the strains. (Strimmer and von Haeseler 1996; Kuraku et al. 2013; Katoh et al. 2019).

### 2.4 Selection of a PGPR Candidate and Testing of Boron (B) Doses

#### 2.4.1 Physical and Chemical Properties of the Soil Used in the Boron Dose Experiment

For the boron dose experiment conducted under greenhouse conditions, the soil material was obtained from the experimental field of Selcuk University campus in Konya. The soil was prepared for analysis by sieving it through a 2 mm mesh. The physical and chemical properties of the experimental soil are summarised in Table 1. The soil exhibits a slightly alkaline reaction, low salt content, very high lime content and low organic matter. It has a clay loam texture. While the soil contains sufficient inorganic nitrogen, its phosphorus and potassium levels are low, although the magnesium content is high. Copper and zinc levels are very low, while iron and manganese concentrations are moderate. The boron content of the soil is adequate.

**TABLE 1:** Physical and chemical properties of the soil used in the boron dose experiment.

Parameters	Results	References
pH	7.75	Jackson (1958)
EC (μS cm^−1^)	149.6	

	**%**	
CaCO_3_	37.70	Hızalan and Ünalan (1966)
Organic Matter	0.75	Smith and Weldon (1941)
Exchangeable Na	1.41	Sağlam (1978)
Clay	37.40	Bouyoucous (1951)
Silt	30.00	
Sand	32.60	
Texture Class	Clay loam	Soil Survey Manual (1951)

	**mg kg^−1^**	
Inorganic Nitrogen (NH_4_‐*N* + NO_3_‐N)	57.80	Bremner (1965)
P	4.76	Olsen (1954)
Ca	4037	US Salinity Lab. Staff (1954)
K	81.20	
Mg	614	
Na	84.20	
Cu	0.15	Lindsay and Norvell (1978)
Fe	2.62	
Mn	2.77	
Zn	0.13	
B	0.59	Cartwright et al. (1983)

#### 2.4.2 Chemical Properties of Barnyard Manure and Peat Used in the Boron Dose Experiment

Manure and peat materials were obtained from commercial suppliers. The manure and peat were prepared for analysis by sieving through a 2 mm mesh. The chemical properties of the organic materials are shown in Table 2. The chemical properties of the barnyard manure and peat, which were incorporated into the soil as part of the growing medium for this experiment, are summarised below. The aim was to create an optimal environment for bacterial growth, which required the chemical analysis of the added materials. Important parameters influencing bacterial development, including pH, Electrical Conductivity (EC), Moisture Content (MC), Carbon (C), Nitrogen (N), C/N ratio and Boron (B) concentration, are outlined below.

**TABLE 2:** Chemical properties of barnyard manure and peat used in the boron dose experiment.

Organic materials	pH	Total macroelements										Total microelements					Water soluable cations			
		mS cm^−1^	%									mg kg^−1^								
		EC	MC	C	N	C/N	Ca	K	Mg	Na	P	B	Cu	Fe	Mn	Zn	Ca	K	Mg	Na
Barnyard manure	8.46	1.96	15.11	42.23	0.51	82.80	2.29	0.56	0.41	0.16	0.24	37.31	35.83	1338.22	90.87	83.07	1542	4709	167	1424
Peat	7.46	1.83	44.10	19.07	0.17	112.18	2.78	0.46	0.62	0.07	0.16	13.47	37.16	19923.63	486.39	77.04	4465	2455	465	669

#### 2.4.3 Testing of Boron (B) Doses for the Selected PGPR Candidate

Biochemical analyses of a bacterial strain tolerant to boron toxicity and salinity were conducted to identify the most suitable PGPR candidate. The effectiveness of this candidate at various boron doses (0, 5, 10, 15, 20, 25 mg kg^−1^ from Etidot‐67) was evaluated in the Computer‐Controlled Research Greenhouse at Selcuk University, Faculty of Agriculture. The study included two application types: presence [B(+)] and absence [B(−)] of bacteria, with a Randomised Plots Two‐Factor Factorial Design and four biological replicates. The growing medium, consisting of soil, peat, manure and sand in a 1:1:1:1 (w/w) ratio, was sieved through a 2 mm mesh. Basic fertilisation was applied before planting to ensure optimal nutrient levels. Wheat ( *Triticum aestivum* L.) seeds were sterilised with sodium hypochlorite, coated with sterile sucrose solution and inoculated with a bacterial suspension (5 × 10^9^ cells ml^−1^). After drying for 24 h, seeds were sown and pots were maintained at field capacity with deionised water, rotated daily to ensure uniform conditions. Morphological parameters (total plant fresh weight, root fresh weight, stem fresh weight, total plant height, root length, plant height, stem dry weight, root dry weight, total plant dry weight, emergence ratio) of wheat plants were evaluated.

### 2.5 Statical Analyses

The results obtained from bacterial laboratory tests were analysed using the JMP 7 statistical software package, whereas the data from greenhouse experiments, conducted using a randomised plot experimental design, were analysed through variance analysis one‐way analysis of variance (ANOVA) with the MINITAB 19 statistical software (Sall et al. 2017; Minitab 2020).

## 3 Results

### 3.1 Biochemical Activities of the Isolated Extremophilic Bacteria and References Bacteria

The biochemical tests conducted play a crucial role in the identification and classification of microorganisms. These tests are essential for understanding the metabolic characteristics and oxygen tolerance of bacteria. By evaluating the bacteria's oxygen utilisation mechanisms and detoxification abilities, these tests help us better understand how environmental microorganisms respond to various conditions.

The study on B toxicity and salinity‐tolerant bacteria isolated from the Eskisehir Kirka Boron mine and salt lake identified nine boron toxicity‐tolerant and six salinity‐tolerant bacteria. All nine boron‐tolerant bacteria from the boron mine were gram‐negative with a bacilli colony morphology. The six salinity‐tolerant bacteria from the salt lake were gram‐positive, with three exhibiting a bacilli colony morphology and the other three a cocci morphology. *E. coli* (ATCC 25922), a gram‐negative bacilli bacterium, was selected as the reference, alongside *P. agglomerans* , also a gram‐negative bacilli bacterium (Table 3).

**TABLE 3:** Biochemical characteristics of bacteria isolated from *Puccinella distans* (Jacq.) Parl. root rhizosphere and reference bacteria.

								TSI test			
	Identification izolates	Gram staining	Colony morphology	Oxidase	Catalase	Urea hyrdolysis	Citrate utilisation test	Acidic	Alkaline	Hydrogen sulphide production	Gas production
Boron toxicity tolerant bacteria	*Sphingobacterium detergens* (1)	(−)	Bacil	−	−	−	−	−	+	−	−
	*Achromobacter spanius*	(−)	Bacil	−	+	−	+	−	+	−	−
	*Pseudomonas extremorientalis*	(−)	Bacil	+	+	−	−	−	+	−	−
	*S. detergens* (2)	(−)	Bacil	−	+	−	±	−	+	−	−
	*Olivibacter soli*	(−)	Bacil	−	−	−	−	+	−	−	−
	*S. detergens* (3)	(−)	Bacil	−	−	−	−	±	−	−	−
	*Pseudomonas poae*	(−)	Bacil	+	+	−	−	+	−	−	−
	*Zhihengliuella halotolerans*	(−)	Bacil	−	+	−	−	−	+	−	−
	*Chryseobacterium* sp.	(−)	Bacil	−	+	**−**	**−**	**+**	**−**	**−**	−
Salinity tolerant bacteria	*Bacillus toyonensis*	(+)	Bacil	−	−	−	−	+	−	−	−
	*Staphylacoccus warneri*	(+)	Coccus	−	+	−	−	+	−	−	−
	*Bacillus muralis*	(+)	Bacil	−	+	−	−	±	−	−	−
	*Staphylacoccus aerus* (1)	(+)	Coccus	−	+	−	−	+	−	−	−
	*S. aerus* (2)	(+)	Coccus	−	+	−	−	−	+	−	−
	*Arthrobacter aurescens*	(+)	Bacil	−	+	**−**	**−**	±	**−**	**−**	−
Reference bacteria	*Escherichia coli* (ATCC 25922)	(−)	Bacil	+	−	−	+	+	−	+	+
	*Pantoea agglomerans*	(−)	Bacil	−	+	−	−	+	−	−	−

The study found that, of the 9 boron toxicity‐tolerant bacteria isolated from the Eskisehir Kirka Boron mine, only 2, *Pseudomonas extremorientalis* and *P. poae* , tested positive in the oxidase test. In contrast, all 6 salinity‐tolerant bacteria from the salt lake tested negative. Among the reference bacteria, *E. coli* (ATCC 25922) tested positive, indicating oxidase enzyme production, whereas *P. agglomerans* tested negative, suggesting the absence of oxidase enzyme production (Table 3).

Among the 9 boron toxicity‐tolerant bacteria isolated from the Eskisehir Kirka Boron mine, 6 bacteria tested positive in the catalase test: *Achromobacter spanius* , *P. extremorientalis* , *Sphingobacterium detergenes* (2), *P. poae* , *Zhihengliuella halotolerans* and *Chryseobacterium* sp. In contrast, *S. detergens* (1), *Olivibacter soli* and *S. detergens* (3) tested negative. Of the six salinity‐tolerant bacteria isolated from the salt lake, five tested positive for catalase: *Staphylococcus warneri* , *Bacillus muralis* , *S. aureus* (1), *S. aureus* (2) and *Arthrobacter aurescens* . *B. toyonensis* tested negative. While *E. coli* (ATCC 25922) also tested negative for catalase, *P. agglomerans* tested positive (Table 3).

None of the Boron Mine, Salt Lake and Reference bacteria could produce the enzyme urease, which is responsible for the hydrolysis of urea to ammonia and carbon dioxide (Table 3).

Among all the boron toxicity‐tolerant and salinity‐tolerant bacterial isolates, only *A. spanius* and *S. detergens* (from the Boron Mining Area) tested positive in the citrate utilisation test. *A. spanius* showed strong positive results, indicating its capacity to utilise citrate as a carbon source, whereas *S. detergens* (2) exhibited weak positive results, suggesting a lower capacity for citrate utilisation. Among the reference bacteria, *E. coli* (ATCC 25922) tested positive, while *P. agglomerans* was negative (Table 3).

The results of the Triple Sugar Iron (TSI) test, hydrogen sulphide (H_2_S) production test and gas production test for bacterial isolates from the Eskisehir Kirka Boron mine, the salt lake and reference bacteria are as follows: Among the nine boron toxicity‐tolerant bacteria from the Eskisehir Kirka Boron mine, *O. soli* , *P. poae* and *Chryseobacterium* sp. exhibited a strong acidic reaction, while *S. detergens* (3) showed a weak acidic reaction. The remaining bacteria displayed an alkaline reaction in the TSI test. Of the six salinity‐tolerant bacteria from the salt lake, five showed an acidic reaction: *B. toyonensis*, *S. warneri* and *S. aureus* (1) displayed a strong acidic reaction, while *B. muralis* and *A. aurescens* showed a weak acidic reaction. *S. aureus* (2) showed an alkaline reaction. All reference bacteria exhibited an acidic reaction. No hydrogen sulphide (H_2_S) production was observed in the test bacteria, but H_2_S production was detected in *E. coli* (ATCC 25922), a reference bacterium, while absent in *P. agglomerans* . Gas production was observed only in *E. coli* (ATCC 25922). In summary, the TSI test showed that some isolates exhibited an acidic reaction, while others were alkaline and hydrogen sulphide and gas production were only observed in *E. coli* (ATCC 25922) (Table 3).

### 3.2 PGPR Activities of the Isolated Extremophilic Bacteria and References Bacteria

Among the 9 boron toxicity‐tolerant bacteria isolated from the Eskisehir Kirka Boron mine, only *O. soli* tested positive in the N‐free test, indicating its ability to fix atmospheric nitrogen, while the remaining bacteria showed negative results. All 6 salinity‐tolerant bacteria from the salt lake also tested negative. The reference *E. coli* (ATCC 25922) also tested negative, whereas *P. agglomerans* yielded a positive result (Table 4).

**TABLE 4:** PGPR characteristics of bacteria isolated from *Puccinella distans* (Jacq.) Parl. root rhizosphere and reference bacteria.

	Identification izolates	N‐free basal medium	P solubulization in NBRIP medium^a^	Indol acetic acid production	Siderophore production	ACC deaminase activity	Ammonium production	Hydrogen cyanide activity
Boron toxicity tolerant bacteria	*Sphingobacterium detergens* (1)	−	−	−	−	−	−	−
	*Achromobacter spanius*	−	−	−	−	−	−	+
	*Pseudomonas extremorientalis*	−	+	−	+	−	−	−
	*S. detergens* (2)	−	−	−	−	−	−	±
	*Olivibacter soli*	+	−	−	−	−	−	−
	*S. detergens* (3)	−	−	−	−	−	−	−
	*Pseudomonas poae*	−	+	−	+	−	+	−
	*Zhihengliuella halotolerans*	−	−	−	−	−	−	−
	*Chryseobacterium* sp.	−	**−**	**−**	**−**	**−**	**−**	**−**
Salinity tolerant bacteria	*Bacillus toyonensis*	−	−	−	−	−	−	−
	*Staphylacoccus warneri*	−	−	−	−	−	−	−
	*Bacillus muralis*	−	−	−	−	−	−	−
	*Staphylacoccus aerus* (1)	−	−	−	−	−	−	−
	*S. aerus* (2)	−	−	−	−	−	−	−
	*Arthrobacter aurescens*	−	**−**	**−**	**−**	**−**	**−**	**−**
Reference bacteria	*Escherichia coli* (ATCC 25922)	−	+	−	+	−	−	+
	*Pantoea agglomerans*	+	−	−	−	*−*	−	−

Abbreviations: +: positive, −: negative, ±: weakly positive.

^a^ The isolates dissolved phosphate or not, and then the amount of solubilization was calculated for those that did.

Only two species from the Kirka mine, *P. extremorientalis* and *P. poae* , tested positive in the NBRIP‐BPB (National Botanical Research Institute's Phosphate Growth Medium‐Bromophenol Blue) test, indicating their ability to solubilise phosphate in the NBRIP medium containing bromophenol blue and tricalcium phosphate. All 6 salinity‐tolerant bacteria tested negative. Both reference bacteria, *E. coli* (ATCC 25922) and *P. agglomerans* , also yielded positive results in the NBRIP‐BPB test (Tables 4 and 5).

**TABLE 5:** Phosphate solubilising potential of tricalcium phosphate and rock phosphate.

P solubilisation in NBRIP medium*		
Phosphate solubilising bacteria	Tricalcium phosphate Ca_3_(PO4)_2_	Rock phosphate
	(mg L^−1^)	
*Pseudomonas extremorientalis*	505.1 ± 5.05^B^	78.05 ± 0.78^B^
*Pseudomonas poae*	299.3 ± 2.99^C^	22.48 ± 0.22^C^
*Escherichia coli* (ATCC 25922)	565.2 ± 5.65^A^	87.08 ± 0.87^A^

*Note:* Letters within the same column represent different groups. Statistical differences were assessed using the Student's *t* test as a multiple comparison test and the LSD was determined (*p* ≤ 0.01). LSD Tricalcium phosphate: 14.24, LSD rock phosphate: 2.12.

* The mean values are presented along with standard deviations.

The bacterial strains were initially evaluated qualitatively for their phosphate solubilising ability on NBRIP‐BPB media. After identifying the phosphorus‐solubilising bacteria, their solubilisation capacity was quantitatively assessed using the ICP‐OES method. The phosphate solubilisation capacity of *P. poae* was given in Table 5.

The results of the Indole Acetic Acid (IAA) production test for all bacterial isolates used in this study, including the reference bacteria, showed negative results. This indicates that none of the tested bacterial species have the ability to produce IAA, a plant growth hormone, under the conditions of the test (Table 4).

The results of the siderophore production test for the bacterial isolates are as follows: Only the two *Pseudomonas* species isolated from the Eskisehir Kirka Boron Mining Area, *P. extremorientalis* and *P. poae* , tested positive for siderophore production. This indicates that these two bacterial strains, but none of the others, are capable of producing siderophores, iron‐chelating compounds that help acquire iron from the environment, facilitating plant uptake. While *E. coli* (ATCC 25922) also yielded a positive result in the siderophore production test, *P. agglomerans* tested negative (Table 4).

All bacterial species and strains, including the reference bacteria, tested negative for ACC deaminase activity. This indicates that these bacteria do not possess ACC deaminase, an enzyme responsible for degrading ACC, a precursor of the plant hormone ethylene. Bacteria with ACC deaminase activity can mitigate the effects of ethylene and promote plant growth under stress conditions. However, the isolated bacteria in this study did not exhibit this activity (Table 4).

Among all the bacteria used in this study, including the reference bacteria, only *P. poae* tested positive in the ammonia production test, indicating that it is the only bacterial isolate capable of producing ammonium as a metabolic byproduct during growth (Table 4).

None of the bacterial isolates from the Boron Mining Area produced HCN during their growth. Among the six bacteria isolated from the salt lake, two species, *S. warneri* and *A. aurescens* , showed positive results in the HCN activity test, indicating their ability to produce HCN as a metabolic byproduct. Both reference bacteria were negative for HCN production (Table 4).

### 3.3 Molecular Characterisation of Extremophilic Bacteria and Reference Bacteria

In this study, 15 extremophilic bacteria were isolated from the rhizosphere of the hyperaccumulator plant *P. distans* , collected from Eskisehir Kirka Boron Mine and Salt Lake. These bacteria were analysed using bioinformatics tools, with their 16S rRNA gene sequences determined and compared to genetic data available in the NCBI database. Nine of the isolated bacteria were tolerant to boron toxicity. Of these, eight were identified at the species level, while one was identified at the genus level. Three strains of *Sphingobacterium* were identified as *S. detergens*, with a 100.00% similarity and their accession number in the NCBI database was MT435019.1. Two species‐level bacteria were identified from the genus *Pseudomonas*: *P. extremorientalis* (100.00% similarity, accession number MT348509.1) and *P. poae* (99.86% similarity, accession number MN173446.1). Other species identified included *A. spanius* (99.93% similarity, accession number CP034689.1), *O. soli* (99.39% similarity, accession number NR_041503.1), *Z. halotolerans* (99.79% similarity, accession number AB778261.1) and a bacterium from the genus *Chryseobacterium* sp. (98.64% similarity, accession number FJ605418.1).

Six bacteria were isolated for their salinity tolerance, all identified at the species level. Three belonged to the genus *Staphylococcus*, namely *S. warneri* (99.14% similarity, accession number MT642942.1) and two strains of *S. aureus* (99.78% and 99.64% similarity, accession number MK809240.1 for both). Two bacteria from the genus *Bacillus* were identified as *B. toyonensis* (98.88% similarity, accession number MN543844.1) and *B. muralis* (99.79% similarity, accession number JQ229793.1). The remaining saline‐tolerant bacterium was identified as *A. aurescens* (98.91% similarity, accession number KT369853.1). As reference bacteria, *E. coli* (ATCC 25922) showed a 100.00% similarity (accession number ASHD01000027.1) and *P. agglomerans* exhibited a 99.36% similarity (accession number OQ848184.1).

### 3.4 Construction of Phylogenetic Trees

**FIGURE 1:**
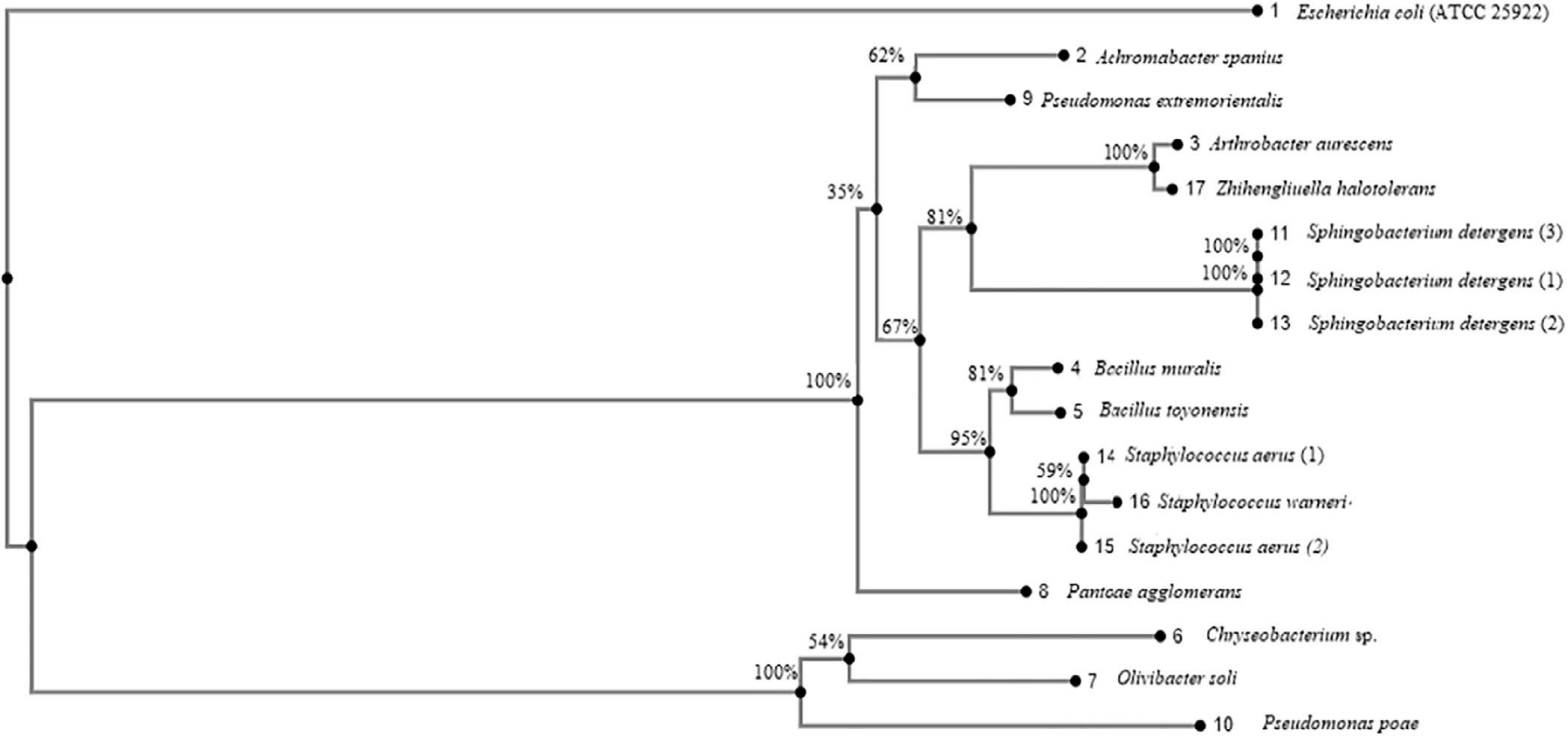
A phylogenetic tree was constructed based on the 16S rRNA gene sequences obtained from bacteria isolated from the rhizosphere of *Puccinella distans* (Jacq.) Parl. The Neighbour‐Joining method was employed, utilising the Jukes–Cantor model. The analysis incorporated a total of 17 representative taxa, consisting of 15 isolated bacterial isolates and two reference bacterial strains ( *Escherichia coli* ATCC 25922 and *Pantoea agglomerans* ). To explore the evolutionary relationships, 1000 bootstrap replicates were generated to assess the stability and reliability of the tree. The percentage of instances in which related taxa formed clusters is indicated adjacent to the branches. This provides a measure of confidence in the accuracy of the branching patterns depicted in the phylogenetic tree. Notably, the phylogenetic tree demonstrates a sequence divergence of 1% among the included sequences.

Figure 1 illustrates the construction of a phylogenetic tree based on 16S rRNA gene sequences derived from bacteria isolated from the root rhizosphere of *Puccinellia distans* (Jacq.) Parl. The tree was generated using the Neighbour–Joining method with the Jukes–Cantor model. A total of 17 representative taxa were included, consisting of 15 isolated bacterial strains and two reference bacterial strains: *E. coli* (ATCC 25922) and *P. agglomerans* .

The phylogenetic tree unfolds in a stepwise manner and is divided into three main groups. The first group is represented by *E. coli* (ATCC 25922), serving as the outgroup and connected to the other two bacterial groups. In the second stage, two distinct openings appear in these groups. Here, *P. agglomerans* separates from its group and links to *E. coli* (ATCC 25922) with 35% similarity. In the third group, *P. poae* diverges from its original group, joining *P. agglomerans* and aligning with *E. coli* (ATCC 25922), confirming the accuracy of the tree's reference bacteria connections.

The third stage sees the separation of the group containing *P. agglomerans* into two subgroups. The first subgroup, with two bacteria, is linked to *P. agglomerans* with 62% similarity, while the second subgroup, containing 10 bacteria, connects to *P. agglomerans* with 67% similarity. In the group where *P. poae* diverges, two bacteria, *Chryseobacterium* sp. and *O. soli* , are present. These bacteria exhibit a 54% similarity to each other before joining *P. poae* , with a 100% similarity.

The fourth stage shows *A. spanius* and *P. extremorientalis* diverging from *P. agglomerans* with a 35% similarity. The remaining subgroup splits into two branches: one with 81% similarity among five bacteria and another with 95% similarity among five bacteria. These branches converge with a 67% similarity, eventually connecting to *P. agglomerans* with 100% similarity.

In the fifth stage, the five‐bacteria branches further divide into sub‐branches, with one containing two bacteria and the other three. These sub‐branches show 81% and 95% similarity, respectively, and are linked to each other with a 67% similarity, converging with the first branch and ultimately attaching to *P. agglomerans* with 100% similarity.

The sixth stage separates taxa within the first node into *A. aurescens* and *Z. halotolerans* , which show a 100% similarity. In the same branch, *S. detergens* (2) exhibits no differentiation from other bacteria, with 100% similarity between them. The second node reveals a bifurcation with *B. muralis* and *B. toyonensis*, which are 81% similar. This node also connects *S. aureus* (2) with other bacteria in the branch, linking them with 100% similarity. The remaining two bacteria in the sub‐branch show a 59% similarity and connect to the group containing *P. agglomerans* .

In the final seventh stage, the two remaining *S. detergens* strains (1 and 3) are linked to *A. aurescens* and *Z. halotolerans* with 81% similarity. Meanwhile, *S. aureus* (1) and *S. warneri* are connected with 59% similarity within the second node, where *S. aureus* (2) is also present. This branch is linked to *B. muralis* and *B. toyonensis* with 95% similarity, completing the comprehensive phylogenetic tree.

### 3.5 Selection of a PGPR Candidate and Testing of Boron (B) Doses

Based on PGPR analyses of bacterial strains tolerant to boron toxicity and salinity, *P. poae* , a Gram‐negative bacterium with several beneficial characteristics, was selected for the boron dose experiment. This bacterium demonstrated effective biochemical responses, including phosphate solubilisation, siderophore production and ammonium generation, alongside its tolerance to acidic boron toxicity. These attributes made *P. poae* the most suitable candidate for further investigation in the context of boron dose tolerance.

#### 3.5.1 Testing of Boron (B) Doses for the Selected PGPR Activities

The effects of boron doses and *P. poae* bacteria [Bacteria (+), Bacteria (−)] treatments on the morphological parameters of bread wheat ( *T. aestivum* L.) are presented in Table 6, including the means of squares and the percentage of variation sources (C.V.).

**TABLE 6:** Means of squares and sources of variation for the effect of *Pseudomonas poae* application [B(+), B(−)] and boron doses on the morphological parameters of bread wheat.

Sources	SD	Means of squares									
		Total plant fresh weight	Root fresh weight	Stem fresh weight	Total plant height	Root lenght	Plant height	Stem dry weight	Root dry weight	Total dry plant weight	Emergance ratio
General	35										
Boron Do$$ses	5	8.56**	1.35*	3.71**	107.89*	115.36*	31.14**	0.078**	0.006**	0.113**	69.57**
Applications	1	48.39**	0.24	55.42**	220.03*	1885.01**	817.01**	0.770**	0.065**	0.387**	617.53**
B Dos.xApp.	5	1.92*	0.15	2.66**	45.49	78.84	46.47**	0.040**	0.003*	0.036**	131.32**
Error	24	0.57	0.34	0.15	37.45	0.34	4.53	0.003	0.001	0.005	0.471
(CV) %		13.41	23.54	12.40	12.79	25.42	8.55	10.16	14.37	8.82	0.73

** *p* < 0.01.

* *p* < 0.05.

The analysis of variance (Table 6) revealed that *P. poae* application [B(+), B(−)] and boron doses significantly affected various morphological parameters of bread wheat ( *T. aestivum* ). Boron doses and applications were statistically significant at the 1% level for total plant fresh weight, stem fresh weight, plant height, stem dry weight, total plant dry weight and germination rate, while the Boron Dose × Application interaction was significant at the 5% level for total plant fresh weight, root dry weight and germination rate. Root fresh weight, total plant height and root length showed significant effects from boron doses and applications, but no significant interaction. The results highlight the beneficial role of *P. poae* in enhancing wheat growth under boron stress, with the interaction between boron doses and bacterial application further influencing plant development.

## 4 Discussion

### 4.1 Biochemical and PGPR Activities of the Isolated Extremophilic Bacteria and the Reference Bacteria

This study reveals diverse biochemical properties among the identified bacteria. Notably, salt‐lake bacteria were Gram‐positive, while boron‐tolerant and reference bacteria were Gram‐negative. Colony morphology showed that *S. aureus* (1), *S. aureus* (2) and *S. warneri* were cocci, while the others were bacilli.

In the oxidase test, *P. extremorientalis* , *P. poae* and *E. coli* (ATCC 25922) exhibited positive results, with *P. extremorientalis* consistently positive. The oxidase response of *P. poae* aligns with Haque et al. (2023), although a weaker reaction was reported by Behrendt et al. (2003). *E. coli* (ATCC 25922), typically oxidase‐negative, showed an unexpected positive result.

Catalase tests were positive for several strains, including *P. extremorientalis* and *P. poae* . The results for *S. detergens* (2) suggest distinct biochemical traits, possibly reflecting different mechanisms. Nitrogen‐fixing capabilities were observed in *O. soli* and *P. agglomerans* , confirming their role in plant growth by converting atmospheric nitrogen into ammonia.

Phosphate solubilisation was evident in *P. extremorientalis* , *P. poae* and *E. coli* (ATCC 25922), with siderophore production aiding plant nutrient uptake. *P. poae* was a notable ammonium producer and *P. extremorientalis* demonstrated phosphate solubilisation, in line with previous findings. Furthermore, bacterial inoculation significantly enhanced tricalcium phosphate solubility in NBRIP medium, outperforming rock phosphate by seven times, consistent with (Ates 2023).

No bacteria showed indole acetic acid production, ACC deaminase activity or urease activity, which is typical for extremophilic bacteria. HCN production was observed in *S. warneri* and *A. aurescens* , suggesting potential as bioagents. Citrate utilisation was noted in *A. spanius* , *S. detergens* (2) and *E. coli* (ATCC 25922), with distinctive traits observed in *S. detergens* (2).

Overall, this study identifies three potential PGPR candidates: *P. extremorientalis* , *P. poae* and *O. soli* . These findings deepen our understanding of the biochemical and PGPR activities of extremophilic bacteria, providing a foundation for future research in this field.

### 4.2 Construction of Phylogenetic Trees

Figure 1 illustrates the phylogenetic tree based on 16S rRNA sequence analysis, highlighting the evolutionary relationships among the bacterial isolates. *P. poae* forms three distinct clusters, including two reference strains, indicating its divergence from other PGPR candidates. *O. soli* , another PGPR candidate, clusters closely with *P. poae* , likely reflecting similarities in their acidic reaction profiles. In contrast, *P. extremorientalis* occupies separate branches, emphasising its distinct phylogenetic position. The tree further demonstrates that all three PGPR candidates and the other isolates occupy unique branches, underscoring their evolutionary diversity.

The observed phylogenetic distribution aligns with prior findings on bacterial adaptation to specific environments. For instance, Mukhtar et al. (2020) identified 24 halophytic bacterial isolates from the root regions of *Salsola* sp. and *Atriplex* sp., with varying PGPR activities based on environmental adaptation, including siderophore production in 21% of the isolates. Similarly, Hassen et al. (2018) described *Pseudomonas rhizophila S211*, a PGPR bacterium isolated from pesticide‐contaminated soils in artichoke cultivation areas, noted for its potential as a biofertilizer, biocontrol agent and bioremediation candidate. These results suggest that the evolutionary adaptations of bacteria to their respective habitats play a critical role in their functional capabilities.

### 4.3 Selection of a PGPR Candidate Was Followed by Testing Boron (B) Doses for Its Activities

Boron is a vital element with significant biological importance, yet its role in bacteria remains underexplored. Research has established its necessity in some unicellular eukaryotes and bacteria, with early studies by Bonilla et al. (1990) indicating boron's potential role in certain *Cyanobacteria*. A notable milestone in boron research was the isolation of *Bacillus boroniphilus* from a boron mine in Hisarcık, Kütahya, by Ahmed et al. (2007). Despite foundational studies on boron‐tolerant bacteria, there is limited exploration of PGPR concerning boron, particularly in agricultural sciences. This study seeks to address this gap and establish a basis for future investigations. Similarly, PGPR confers plant tolerance to abiotic stresses such as salinity and high boron (B) by limiting uptake of toxic ions and increasing antioxidant production and *B. pumilus* inoculation increased antioxidant capacity in rice plants under salinity and boron stress (Khan et al. 2016).

In related research, Özdoğan et al. (2022) evaluated 69 bacterial strains representing 12 genera for their nitrogen fixation and phosphate solubilisation potential through a pot experiment with wheat. Treatments included a control, chemical fertilisers and seven bacterial isolates. Chemical fertilisers resulted in the highest stem fresh and dry weights and plant height, while the C1/7 isolate produced comparable outcomes, underscoring its potential as an eco‐friendly alternative. Similarly, PGPR applications, including strains such as *Bacillus* OSU‐142 and *Azospirillum brasilense* sp.245, have been shown to optimise wheat and barley yields while enhancing plant growth and enzymatic activities through nitrogen fixation and phosphate solubilisation, with species‐specific effect (Cakmakci et al. 2007, 2014). Further studies by (Mehboob et al. 2021) investigated the co‐application of boron‐tolerant bacteria (BTB) with synthetic boron fertilisers to enhance chickpea yield and grain‐boron concentration. *Bacillus* sp. MN54 inoculation significantly improved root growth, nodulation and yield, with the highest grain‐boron concentration observed at 0.75 mg B kg^−1^ boron. The combination of *Bacillus* sp. MN54 and 0.25 mg B kg^−1^ boron proved most effective for promoting growth and biofortification in chickpeas. Similarly, Sen et al. (2022) studied *Lysinibacillus* sp. OL1 and *Enterococcus* sp. OL5, two boron‐tolerant bacterial strains, which demonstrated resistance and growth promotion at high boron concentrations. Their ability to modulate boron uptake and transport mechanisms, supported by genomic analysis, suggests their potential in reducing boron toxicity and enhancing soil microbiota bioavailability, making them valuable as biofertilisers.

## 5 Conclusion

This study aimed to isolate, molecularly characterise and determine the phylogenetic relationships of extremophilic bacteria tolerant to boron toxicity and salinity and to conduct PGPR analyses on them. The bacteria were isolated from the rhizosphere of *P. distans* , a plant native to the Eskisehir Kirka Boron Mine and Salt Lake (local name Tuz Gölü) in the Central Anatolian Region of Türkiye. Three PGPR candidates were identified: *P. extremorientalis* , *P. poae* and *O. soli* , all of which exhibited tolerance to boron toxicity. Among these, *P. poae* demonstrated the most significant biochemical responses. In greenhouse trials under conditions of boron toxicity on bread wheat ( *T. aestivum* ), *P. poae* showed promising results, indicating its potential as an extremophilic microbial fertiliser for cultivation in boron‐toxic environments. Future recommendations include exploring the use of such extremophilic microbial fertilisers for managing abiotic stress conditions. These bacterial species, either individually or in combination, could serve as effective microbial fertiliser candidates for enhancing agricultural productivity in boron‐toxic areas. This has significant implications for regions where boron toxicity reduces agricultural yield, including significant parts of Türkiye and other similar agroecological areas worldwide. Further recommendations include establishing a cultural collection of extremophilic bacteria, evaluating their efficacy in various trials such as jar, beaker, greenhouse and field studies as PGPR candidates and comparing their performance within the soil microbiome through microbiome studies. Additionally, investigations on the application methods, bacterial compatibility with suitable carriers, interactions among bacteria and their impact on crop yield should be conducted. This study provides a foundational framework for future research, highlighting differences and potential applications in the field.

## Author Contributions

Noyan Eken conducted the research and wrote the article. Ozgür Ates, Ramazan Cakmakci, Ummahan Çetin Karaca and Sait Gezgin assisted with the research and the editing of the article. Erdogan Esref Hakki served as the supervising faculty member, overseeing the progression and finalization of the study and the editing of the article.

## Conflicts of Interest

The authors declare no conflicts of interest.

## Data Availability

The data that support the findings of this study are available on request from the corresponding author. The data are not publicly available due to privacy or ethical restrictions.

## References

[emi470105-bib-0001] Agami, R. A. , H. A. Ghramh , and M. Hasheem . 2017. “Seed Inoculation With *Azospirillum lipoferum* Alleviates the Adverse Effects of Drought Stress on Wheat Plants.” Journal of Applied Botany and Food Quality 90: 165–173.

[emi470105-bib-0002] Ahmed, I. , and T. Fujiwara . 2010. “Mechanism of Boron Tolerance in Soil Bacteria.” Canadian Journal of Microbiology 56: 22–26. 20130690 10.1139/w09-106

[emi470105-bib-0003] Ahmed, I. , A. Yokota , and T. Fujiwara . 2007. “A Novel Highly Boron Tolerant Bacterium, *Bacillus boroniphilus* sp. Nov., Isolated From Soil, That Requires Boron for Its Growth.” Extremophiles 11: 217–224. 17072687 10.1007/s00792-006-0027-0

[emi470105-bib-0004] Ates, Ö. 2023. “Phosphate Solubilizing Bacteria Isolation Medium: Rock Phosphate or Tricalcium Phosphate?” Geomicrobiology Journal 40: 751–755.

[emi470105-bib-0005] Baati, H. , R. Amdouni , N. Gharsallah , A. Sghir , and E. Ammar . 2010. “Isolation and Characterization of Moderately Halophilic Bacteria From Tunisian Solar Saltern.” Current Microbiology 60: 157–161. 19826862 10.1007/s00284-009-9516-6

[emi470105-bib-0006] Bakker, A. W. , and B. Schippers . 1987. “Microbial Cyanide Production in the Rhizosphere in Relation to Potato Yield Reduction and Pseudomonas Spp‐Mediated Plant Growth‐Stimulation.” Soil Biology and Biochemistry 19: 451–457.

[emi470105-bib-0007] Behrendt, U. , A. Ulrich , and P. Schumann . 2003. “Fluorescent Pseudomonas Associated With the Phyllosphere of Grasses; *Pseudomonas trivialis* sp. Nov., *Pseudomonas poae* sp. nov. and *Pseudomonas congelans* sp. Nov.” International Journal of Systematic and Evolutionary Microbiology 53: 1461–1469. 13130034 10.1099/ijs.0.02567-0

[emi470105-bib-0008] Bharucha, U. , K. Patel , and U. B. Trivedi . 2013. “Optimization of Indole Acetic Acid Production by *Pseudomonas putida* UB1 and Its Effect as Plant Growth‐Promoting Rhizobacteria on Mustard ( *Brassica nigra* ).” Agricultural Research 2: 215–221.

[emi470105-bib-0009] Bishnoi, U. 2015. “PGPR Interaction: An Ecofriendly Approach Promoting the Sustainable Agriculture System.” In Advances in Botanical Research, edited by H. Bais and J. Sherrier , 81–113. Academic Press.

[emi470105-bib-0010] Bonilla, I. , M. Garcia‐Gonzalez , and P. Mateo . 1990. “Boron Requirement in Cyanobacteria: Its Possible Role in the Early Evolution of Photosynthetic Organisms.” Plant Physiology 94: 1554–1560. 16667889 10.1104/pp.94.4.1554 PMC1077420

[emi470105-bib-0012] Bouyoucous, G. J. 1951. “Recalibration of the Hydrometer Method for Making Mechanical Analysis of Soils.” Agronomy 43: 434–438.

[emi470105-bib-0013] Brdar‐Jokanović, M. 2020. “Boron Toxicity and Deficiency in Agricultural Plants.” International Journal of Molecular Sciences 21: 1424. 32093172 10.3390/ijms21041424 PMC7073067

[emi470105-bib-0014] Brdar‐Jokanović, M. , I. Maksimović , M. Kraljević‐Balalić , T. Zeremski‐Škorić , A. Kondić‐Špika , and B. Kobiljski . 2013. “Boron Concentration vs. Content as Criterion for Estimating Boron Tolerance in Wheat.” Journal of Plant Nutrition 36: 470–480.

[emi470105-bib-0015] Bremner, J. 1965. “Inorganic Forms of Nitrogen.” In Methods of Soil Analysis. Part 2. Agron. Monogr. 9, edited by C. A. Black , 1179–1237. ASA.

[emi470105-bib-0016] Cabaj, A. , and A. Kosakowska . 2009. “Iron‐Dependent Growth of and Siderophore Production by Two Heterotrophic Bacteria Isolated From Brackish Water of the Southern Baltic Sea.” Microbiological Research 164: 570–577. 17689229 10.1016/j.micres.2007.07.001

[emi470105-bib-0017] Cakmakci, R. , M. Erat , Ü. Erdoğan , and M. F. Dönmez . 2007. “The Influence of Plant Growth–Promoting Rhizobacteria on Growth and Enzyme Activities in Wheat and Spinach Plants.” Journal of Plant Nutrition and Soil Science 170: 288–295.

[emi470105-bib-0018] Cakmakci, R. , M. Turan , M. Gulluce , and F. Sahin . 2014. “Rhizobacteria for Reduced Fertilizer Inputs in Wheat (*Triticum aestivum* spp. Vulgare) and Barley (*Hordeum vulgare*) on Aridisols in Turkey.” International Journal of Plant Production 8, no. 2: 163–182.

[emi470105-bib-0019] Cappuccino, J. G. , and N. Sherman . 1996. Instructor's Guide for Microbiology: A Laboratory Manual. Benjamin/Cummings Publishing Company.

[emi470105-bib-0074] Cartwright, B. , K. G. Tiller , B. A. Zarcinas , and L. R. Spouncer . 1983. “The Chemical Assessment of the Boron Status of Soils.” Soil Research 21, no. 3: 321–332.

[emi470105-bib-0020] Chebotar, V. , E. Chizhevskaya , O. Khonina , et al. 2023. “Biotechnological Potential of Galophytes and Their Microbiomes for Agriculture in Russia and Kazakhstan.” Russian Journal of Plant Physiology 70: 183.

[emi470105-bib-0021] Cheng, Y. , X. Xie , X. Wang , L. Zhu , Q.‐S. Qiu , and X. Xu . 2023. “Effects of the Salt‐Tolerant Gramineous Forage *Echinochloa frumentacea* on Biological Improvement and Crop Productivity in Saline–Alkali Land on the Hetao Ningxia Plain in China.” Sustainability 15: 5319.

[emi470105-bib-0022] Chester, B. 1979. “Semiquantitative Catalase Test as an Aid in Identification of Oxidative and Nonsaccharolytic Gram‐Negative Bacteria.” Journal of Clinical Microbiology 10: 525–528. 393719 10.1128/jcm.10.4.525-528.1979 PMC273208

[emi470105-bib-0023] Collee, J. G. , R. Miles , and B. Watt . 1996. “Tests for Identification of Bacteria.” In Mackie and McCartney Practical Medical Microbiology, edited by J. G. Collee , A. G. Fraser , B. P. Marmion , and A. Simmons , vol. 14, 131–149. Churchill Livingstone.

[emi470105-bib-0024] Collins, C. , P. M. Lyne , J. M. Grange , and J. O. Falkinham . (Eds.). 2004. Collins and Lyne's Microbiological Methods, 8th ed, 144–155. Arnold.

[emi470105-bib-0025] de Freitas, J. , M. Banerjee , and J. Germida . 1997. “Phosphate‐Solubilizing Rhizobacteria Enhance the Growth and Yield but Not Phosphorus Uptake of Canola (*Brassica napus* L.).” Biology and Fertility of Soils 24: 358–364.

[emi470105-bib-0026] Dworkin, M. , and J. Foster . 1958. “Experiments With Some Microorganisms Which Utilize Ethane and Hydrogen.” Journal of Bacteriology 75: 592–603. 13538930 10.1128/jb.75.5.592-603.1958 PMC290115

[emi470105-bib-0027] Eken, N. , and E. E. Hakki . 2024. “Plant‐Microbiota Interactions Under Drought and Salinity Stresses: A Mini Review.” Agricultural Research & Technology: Open Access Journal 28: 3.

[emi470105-bib-0028] Gregory, P. J. 2006. “Roots, Rhizosphere and Soil: The Route to a Better Understanding of Soil Science?” European Journal of Soil Science 57: 2–12.

[emi470105-bib-0029] Hakki, E. , A. Pandey , M. Khan , et al. 2019. “ *Puccinellia distans*—A potential Plant to Reveal Boron Toxicity and Salt Tolerance Mechanisms.” Journal of Biotechnology 305: S19. 10.1016/j.jbiotec.2019.05.078.

[emi470105-bib-0030] Hamurcu, M. , E. E. Hakki , T. D. Sert , et al. 2016. “Extremely High Boron Tolerance in *Puccinellia Distans* (Jacq.) Parl. Related to Root Boron Exclusion and a Well‐Regulated Antioxidant System.” Zeitschrift Fur Naturforschung Section C—A Journal of Biosciences 71, no. 7‐8: 273–285. 10.1515/znc-2015-0226. 27356235

[emi470105-bib-0031] Han, J. , L. Sun , X. Dong , et al. 2005. “Characterization of a Novel Plant Growth‐Promoting Bacteria Strain *Delftia tsuruhatensis* HR4 Both as a Diazotroph and a Potential Biocontrol Agent Against Various Plant Pathogens.” Systematic and Applied Microbiology 28: 66–76. 15709367 10.1016/j.syapm.2004.09.003

[emi470105-bib-0032] Haque, M. M. , M. Khatun , M. K. Mosharaf , A. Rahman , M. A. Haque , and K. Nahar . 2023. “Biofilm Producing Probiotic Bacteria Enhance Productivity and Bioactive Compounds in Tomato.” Biocatalysis and Agricultural Biotechnology 50: 102673.

[emi470105-bib-0033] Harley, J. , and L. Prescott . 2002. Laboratory Exercises in Microbiology. McGraw—Hill.

[emi470105-bib-0034] Hassen, W. , M. Neifar , H. Cherif , et al. 2018. “Pseudomonas Rhizophila S211, a New Plant Growth‐Promoting Rhizobacterium With Potential in Pesticide‐Bioremediation.” Frontiers in Microbiology 9: 34. 29527191 10.3389/fmicb.2018.00034 PMC5829100

[emi470105-bib-0035] Hızalan, E. , and H. Ünalan . 1966. “Toprakta Önemli Kimyasal Analizler.” Ankara Üniversitesi Ziraat Fakültesi Yayın No: 273, Ankara. 466.

[emi470105-bib-0036] Jackson, M. 1958. Soil Chemical Analysis. Vol. 183. Prentice‐Hall, Inc.

[emi470105-bib-0037] Jha, A. K. , S. Kumar , and A. Kumar . 2015. “Zhihengliuella Somnathii sp. Nov., a New Halotolerant Bacterium Isolated From Saline Soil.” International Journal of Systematic and Evolutionary Microbiology 65: 3820–3824. 10.1099/ijsem.0.000391 26297009

[emi470105-bib-0038] Jha, C. K. , and M. Saraf . 2015. “Plant Growth Promoting Rhizobacteria (PGPR): A Review.” Journal of Agricultural Research and Development 5: 108–119.

[emi470105-bib-0039] Katoh, K. , J. Rozewicki , and K. D. Yamada . 2019. “MAFFT Online Service: Multiple Sequence Alignment, Interactive Sequence Choice and Visualization.” Briefings in Bioinformatics 20: 1160–1166. 28968734 10.1093/bib/bbx108 PMC6781576

[emi470105-bib-0040] Khan, A. L. , B. A. Halo , A. Elyassi , et al. 2016. “Indole Acetic Acid and ACC Deaminase From Endophytic Bacteria Improves the Growth of *Solanum lycopersicum* .” Electronic Journal of Biotechnology 21: 58–64.

[emi470105-bib-0041] Kopittke, P. M. , N. W. Menzies , P. Wang , B. A. McKenna , and E. Lombi . 2019. “Soil and the Intensification of Agriculture for Global Food Security.” Environment International 132: 105078. 31400601 10.1016/j.envint.2019.105078

[emi470105-bib-0042] Kuraku, S. , C. M. Zmasek , O. Nishimura , and K. Katoh . 2013. “A Leaves Facilitates On‐Demand Exploration of Metazoan Gene Family Trees on MAFFT Sequence Alignment Server With Enhanced Interactivity.” Nucleic Acids Research 41: W22–W28. 23677614 10.1093/nar/gkt389 PMC3692103

[emi470105-bib-0071] Lindsay, W. L. , and W. Norvell . 1978. “Development of a DTPA Soil Test for Zinc, Iron, Manganese, and Copper.” Soil Science Society of America Journal 42, no. 3: 421–428.

[emi470105-bib-0043] Louden, B. C. , D. Haarmann , and A. M. Lynne . 2011. “Use of Blue Agar CAS Assay for Siderophore Detection.” Journal of Microbiology & Biology Education 12: 51–53. 23653742 10.1128/jmbe.v12i1.249 PMC3577196

[emi470105-bib-0044] Malhi, Y. , J. Franklin , N. Seddon , et al. 2020. Climate Change and Ecosystems: Threats, Opportunities and Solutions, 20190104. Royal Society. 10.1098/rstb.2019.0104 PMC7017779 31983329

[emi470105-bib-0045] Mehboob, N. , M. Hussain , W. A. Minhas , et al. 2021. “Soil‐Applied Boron Combined With Boron‐Tolerant Bacteria (Bacillus sp. mn54) Improve Root Proliferation and Nodulation, Yield and Agronomic Grain Biofortification of Chickpea ( *Cicer arietinum* L.).” Sustainability 13: 9811.

[emi470105-bib-0046] Mehta, S. , and C. S. Nautiyal . 2001. “An Efficient Method for Qualitative Screening of Phosphate‐Solubilizing Bacteria.” Current Microbiology 43: 51–56. 11375664 10.1007/s002840010259

[emi470105-bib-0047] Minitab LLC . 2020. “Minitab Statistical Software, Version 19.” 2022, State College, PA, USA.

[emi470105-bib-0048] Mukhtar, S. , M. Zareen , Z. Khaliq , S. Mehnaz , and K. Malik . 2020. “Phylogenetic Analysis of Halophyte‐Associated Rhizobacteria and Effect of Halotolerant and Halophilic Phosphate‐Solubilizing Biofertilizers on Maize Growth Under Salinity Stress Conditions.” Journal of Applied Microbiology 128: 556–573. 31652362 10.1111/jam.14497

[emi470105-bib-0049] Olsen, S. R. 1954. Estimation of Available Phosphorus in Soils by Extraction With Sodium Bicarbonate. US Department of Agriculture.

[emi470105-bib-0050] Özdoğan, D. K. , N. Akçelik , and M. Akçelik . 2022. “Genetic Diversity and Characterization of Plant Growth‐Promoting Effects of Bacteria Isolated From Rhizospheric Soils.” Current Microbiology 79: 132. 35290524 10.1007/s00284-022-02827-3

[emi470105-bib-0073] Penrose, D. M. , and B. R. Glick . 2003. “Methods for Isolating and Characterizing ACC Deaminase‐Containing Plant Growth‐Promoting Rhizobacteria.” Physiologia Plantarum 118, no. 1: 10–15. 12702008 10.1034/j.1399-3054.2003.00086.x

[emi470105-bib-0051] Princi, M. P. , A. Lupini , F. Araniti , et al. 2015. Boron Toxicity and Tolerance in Plants: Recent Advances and Future Perspectives, 115–147. Elsevier. 10.1016/B978-0-12-803158-2.00005-9.

[emi470105-bib-0052] Sağlam, M. 1978. Toprak kimyası Tatbikat Notları. Atatürk Üniversitesi Ziraat Fakültesi Toprak Bölümü.

[emi470105-bib-0053] Sall, J. , M. L. Stephens , A. Lehman , and S. Loring . 2017. JMP Start Statistics: A Guide to Statistics and Data Analysis Using JMP. Sas Institute.

[emi470105-bib-0054] Sarker, A. , and J. Al‐Rashid . 2013. “Analytical Protocol for Determination of Indole 3 Acetic Acid (IAA) Production by Plant Growth Promoting Bacteria (PGPB).” Technical Report of Quantification of IAA, Microbes, September 3–5.

[emi470105-bib-0055] Schwyn, B. , and J. Neilands . 1987. “Universal Chemical Assay for the Detection and Determination of Siderophores.” Analytical Biochemistry 160: 47–56. 2952030 10.1016/0003-2697(87)90612-9

[emi470105-bib-0056] Sen, S. , N. Mondal , W. Ghosh , and R. Chakraborty . 2022. “Inducible Boron Resistance via Active Efflux in Lysinibacillus and Enterococcus Isolates From Boron‐Contaminated Agricultural Soil.” Biometals 35: 215–228. 35037170 10.1007/s10534-021-00359-0

[emi470105-bib-0057] Smith, H. W. , and M. Weldon . 1941. “A Comparison of Some Methods for the Determination of Soil Organic Matter.” Soil Science Society of America Journal 5: 177–182.

[emi470105-bib-0058] Sneath, P. , J. Abbott , and A. Cunliffe . 1951. “Bacteriology of Erysipeloid.” British Medical Journal 2: 1063. 14869814 10.1136/bmj.2.4739.1063 PMC2070599

[emi470105-bib-0059] Sorokin, D. Y. , T. Tourova , E. Galinski , C. Belloch , and B. Tindall . 2006. “Extremely Halophilic Denitrifying Bacteria From Hypersaline Inland Lakes, *Halovibrio denitrificans* sp. Nov. and *Halospina denitrificans* Gen. Nov., sp. Nov., and Evidence That the Genus Name Halovibrio Fendrich 1989 With the Type Species *Halovibrio variabilis* Should Be Associated With DSM 3050.” International Journal of Systematic and Evolutionary Microbiology 56: 379–388. 16449444 10.1099/ijs.0.63964-0

[emi470105-bib-0060] Strimmer, K. , and A. von Haeseler . 1996. “Accuracy of Neighbor Joining for n‐Taxon Trees.” Systematic Biology 45: 516–523.

[emi470105-bib-0061] Timmusk, S. , B. Nicander , U. Granhall , and E. Tillberg . 1999. “Cytokinin Production by *Paenibacillus polymyxa* .” Soil Biology and Biochemistry 31: 1847–1852.

[emi470105-bib-0062] Torun, A. A. , A. Yazici , H. Erdem , and İ. Çakmak . 2006. “Genotypic Variation in Tolerance to Boron Toxicity in 70 Durum Wheat Genotypes.” Turkish Journal of Agriculture and Forestry 30: 49–58.

[emi470105-bib-0063] United States . 1951. “Bureau of Plant Industry, Soils, and Agricultural Engineering. Soil Survey Manual.” No. 18. Agricultural Research Administration, US Department of Agriculture, 1951. https://books.google.com.tr/books?hl=tr&lr=&id=YvXWw7BcxqYC&oi=fnd&pg=PP9&dq=US+Department+of+Agriculture.+1951.+Soil+Survey+Manual.+Bureau+of+Plant+Industry+Soils+Agricultural+Engineering+United+States.+Science+Education+Administration:+Science+Education+Administration:+US+Department+of+Agriculture.&ots=7‐f7fcu1PI&sig=G8IidhQZXplfNaxFECdP7r8zTsk&redir_esc=y#v=onepage&q=US%20Department%20of%20Agriculture.%201951.%20Soil%20Survey%20Manual.%20Bureau%20of%20Plant%20Industry%20Soils%20Agricultural%20Engineering%20United%20States.%20Science%20Education%20Administration%3A%20Science%20Education%20Administration%3A%20US%20Department%20of%20Agriculture.&f=false.

[emi470105-bib-0064] US Salinity Laboratory Staff . 1954. Diagnosis and Improvement of Saline and Alkali Soils. Agriculture Handbook 60, 83–100. US Salinity Laboratory Staff.

[emi470105-bib-0065] Valsalakumar, N. , J. Ray , and V. Potty . 2007. “Arbuscular Mycorrhizal Fungi Associated With Green Gram in South India.” Agronomy Journal 99: 1260–1264.

[emi470105-bib-0066] Vera, A. , J. Moreno , J. Siles , et al. 2021. “Interactive Impacts of Boron and Organic Amendments in Plant‐Soil Microbial Relationships.” Journal of Hazardous Materials 408: 124939. 33383449 10.1016/j.jhazmat.2020.124939

[emi470105-bib-0067] Yang, C. , W. Zhao , Y. Wang , L. Zhang , S. Huang , and J. Lin . 2020. “Metabolomics Analysis Reveals the Alkali Tolerance Mechanism in *Puccinellia tenuiflora* Plants Inoculated With Arbuscular Mycorrhizal Fungi.” Microorganisms 8: 327. 32110985 10.3390/microorganisms8030327 PMC7142761

[emi470105-bib-0068] Zhang, X. , Y. Mi , H. Mao , S. Liu , L. Chen , and F. Qin . 2020. “Genetic Variation in ZmTIP1 Contributes to Root Hair Elongation and Drought Tolerance in Maize.” Plant Biotechnology Journal 18: 1271–1283. 31692165 10.1111/pbi.13290 PMC7152618

[emi470105-bib-0069] Zhao, Q. , J. Li , Z. Dai , C. Ma , H. Sun , and C. Liu . 2019. “Boron Tolerance and Accumulation Potential of Four Salt‐Tolerant Plant Species.” Scientific Reports 9: 6260. 31000729 10.1038/s41598-019-42626-8 PMC6472400

